# S100A10 knockdown exacerbates phenylephrine-induced cardiomyocyte hypertrophy via modulating mitochondrial oxidative phosphorylation

**DOI:** 10.3389/fgene.2025.1610008

**Published:** 2025-10-22

**Authors:** Feixue Xu, Yajie Chen, Man Xu, Dan Li, Yinshan Lu, Meng Zhang, Jiahao Li, Wanyi Li, Yingying Guo

**Affiliations:** ^1^ Department of Cardiology, Renmin Hospital of Wuhan University, Wuhan, China; ^2^ Hubei Key Laboratory of Metabolic and Chronic Diseases, Wuhan, China

**Keywords:** cardiomyocyte hypertrophy, S100A10, AnxA2, mTOR/4E-BP signaling pathway, mitochondria

## Abstract

**Background:**

Mitochondrial dysfunction is a well-established hallmark of pathological cardiac hypertrophy, though its underlying mechanisms are not fully understood. S100A10, a calcium-binding protein, participates in diverse cellular processes, including the regulation of mTOR signaling and mitochondrial function. This study aims to investigate the role and mechanistic basis of S100A10 in phenylephrine (PE)-induced cardiomyocyte hypertrophy.

**Methods:**

Primary neonatal rat cardiomyocytes (NRVMs) were treated with phenylephrine (PE) to induce hypertrophy. S100A10 expression was modulated by siRNA knockdown. The interaction between S100A10 and ANXA2 was confirmed by co-immunoprecipitation. mTOR pathway activation was analyzed by Western blotting. Mitochondrial function was assessed by measuring the expression of electron transport chain complexes, mitochondrial membrane potential using JC-1 staining, and mitochondrial oxidative stress using MitoSOX.

**Results:**

S100A10 expression was significantly upregulated in hypertrophic murine hearts. We further demonstrated that S100A10 interacts with ANXA2 to activate the mTOR/4E-BP signaling pathway. Knockdown of S100A10 in NRVMs suppressed the expression of mitochondrial respiratory chain proteins, impaired oxidative phosphorylation activity, and reduced mitochondrial membrane potential and ATP production.

**Conclusion:**

These findings indicate that downregulation of S100A10 exacerbates PE-induced cardiomyocyte hypertrophy and uncover a novel function of S100A10 in modulating mitochondrial respiratory chain protein levels, potentially through the mTOR/4E-BP pathway. This may provide a theoretical basis for future therapeutic strategies.

## 1 Introduction

Heart failure (HF), a multifaceted clinical syndrome, is a major cause of morbidity and mortality worldwide (Savarese et al., 2023). Under conditions of prolonged cardiac stress, such as chronic hypertension or other stimulation, early-stage cardiac hypertrophy serves as an adaptive mechanism to preserve heart function (Zeng et al., 2020). However, with sustained stress, this compensatory response transitions to a pathological state, characterized by altered gene transcription, extracellular matrix deposition (fibrosis), and increased cardiomyocyte size, ultimately impairing cardiac function and leading to heart failure (Valenzuela et al., 2016). Activation of the mTOR pathway has been shown to ameliorate pathological cardiac hypertrophy by improving protein turnover and mitochondrial function. Dysregulation of the mTOR pathway has been implicated in various cardiovascular diseases, including heart failure (Park et al., 2018). Therefore, a deeper understanding of the interactions between mTOR signaling and other molecular pathways could reveal the mechanisms driving cardiac remodeling and help identify new targets for therapeutic interventions.

S100A10 (also known as p11) is a member of the S100 protein family of calcium-binding proteins; however, unlike other members, S100A10 has lost its ability to bind calcium (Bharadwaj A. et al., 2021). It is ubiquitously expressed in various tissues throughout the human body, with notable abundance in the nervous and muscular systems, and can participate in multiple cellular physiological processes, such as membrane transport, cytoskeletal dynamics, and fibrinolysis (Okura et al., 2023). S100A10 exerts its functional effects largely through its interaction with annexin A2 (ANXA2), forming a heterotetramer complex. This complex plays a critical role in processes such as plasminogen activation and angiogenesis, highlighting its importance in cellular remodeling and cardiovascular physiology (Bharadwaj A. G. et al., 2021). Recent studies have demonstrated that S100A10 interacts with annexin A2 (ANXA2) to activate the mTOR pathway, thereby promoting tumor glycolysis and driving malignant tumor progression (Li et al., 2020). These findings underscore the therapeutic potential of targeting S100A10 in cardiac remodeling and further establish its role in this process. S100A10 thus represents a promising therapeutic target for cardiac hypertrophy and heart failure, though its precise mechanistic contributions warrant deeper investigation.

In this study, we identified differential expression of S100A10 both in gene sets derived from TAC-induced cardiac hypertrophy, dilated cardiomyopathy and ischemic cardiomyopathy. *In vitro*, we demonstrated S100A10 exerts its effects by binding to ANXA2, activating the mTOR/4E-BPs signaling pathways, and regulating mitochondrial oxidative phosphorylation activity. Collectively, our findings elucidate the critical role of S100A10 in modulating mitochondrial oxidative phosphorylation activity and establish it as a potential therapeutic target for phenylephrine-induced cardiomyocyte hypertrophy.

## 2 Materials and methods

### 2.1 Materials

All chemicals and reagents were obtained from commercial sources. Cell culture media (DMEM) and fetal bovine serum (FBS) were purchased from Gibco (Thermo Fisher Scientific, United States). RNA extraction and cDNA synthesis kits were acquired from Roche (Switzerland). Antibodies for Western blotting and co-immunoprecipitation were obtained from Cell Signaling Technology (United States). S100a10 siRNA and control siRNA were sourced from Invitrogen (Thermo Fisher Scientific, United States). Seahorse XF assay kits were from Agilent Technologies (United States). Cell culture consumables including 6-well plates, 96-well plates, and related materials were purchased from Servicebio (Wuhan, China).

### 2.2 Identification of differentially expressed genes (DEGs)

Public datasets (accession numbers: GSE5500, GSE116250, and GSE36961) were downloaded from the Gene Expression Omnibus (GEO) database. Differential gene expression analysis was performed using the DESeq2 package (version 1.38.3) in R (version 4.4.1), with significance thresholds set at adjusted p-value <0.05 and absolute log2 fold change >0.5 for both upregulated and downregulated genes.

### 2.3 Isolation and culture of neonatal rat ventricular myocytes (NRVMs)

Ventricular cardiomyocytes were isolated from 1-2-day-old Sprague Dawley rats by enzymatic digestion using collagenase type II (0.5 mg/mL) and pancreatin (0.6 mg/mL) (Worthington Biochemical Corp, NJ). Isolated NRVMs were cultured in DMEM/F12 medium supplemented with 15% FBS and maintained at 37 °C in a 5% CO2 humidified incubator. To induce hypertrophy, cells were treated with 20 μM phenylephrine (PE; Sigma-Aldrich, United States) for 48 h.

### 2.4 RNA interference

NRVMs were transfected with either S100a10-specific siRNA (forward: 5′-CGG​GGC​CCA​GGT​TTC​GAC​AG-3'; reverse: 5′-CCC​GTT​CCA​TGA​GCA​CTC​TCA​GGT-3′) or non-targeting control siRNA (20 nM final concentration) using Lipofectamine™ 2000 (Thermo Fisher Scientific, United States) according to the manufacturer’s protocol. Twelve hours post-transfection, cells were treated with PE to induce hypertrophy. Cells were harvested 48 h after transfection for subsequent analyses.

### 2.5 Quantitative real-time PCR and western blotting

Total RNA was extracted using RNA extraction kits (Roche, Switzerland), and cDNA was synthesized using cDNA synthesis kits (Roche, Switzerland). qRT-PCR was performed using SYBR Green PCR Master Mix (Roche, Switzerland) with GAPDH as the internal control.

For Western blotting, proteins were extracted using RIPA buffer (Thermo Fisher Scientific, United States) containing protease and phosphatase inhibitors. Protein concentrations were determined by BCA assay (Thermo Fisher Scientific, United States). Equal protein amounts (20–40 μg) were separated by 10% SDS-PAGE and transferred to PVDF membranes (Millipore, United States). Membranes were blocked with 5% non-fat milk in TBS-T, then incubated overnight at 4 °C with primary antibodies against S100A10, ANXA2, or β-tubulin, followed by HRP-conjugated secondary antibodies (Cell Signaling Technology, United States). Protein bands were visualized using ECL (Thermo Fisher Scientific, United Statesa) and quantified using ImageJ software.

### 2.6 Measurement of mitochondrial superoxide levels

NRVMs were incubated with 5 μM MitoSOX Red (Beyotime, Jiangsu, China) for 30 min at 37 °C in the dark. After washing, mitochondrial superoxide levels were assessed by fluorescence microscopy (Olympus, Japan).

### 2.7 Seahorse metabolic assay

Cellular metabolism was analyzed using the Seahorse XF24 Analyzer (Agilent Technologies, United States). NRVMs were seeded at 70% confluency in XF24 plates and treated with either 20 μM PE or PBS (control) for 48 h. The oxygen consumption rate (OCR) was measured under basal conditions and after sequential injection of mitochondrial inhibitors (oligomycin, FCCP, and antimycin A). Data were analyzed using Seahorse Wave Software (Agilent Technologies, United States).

### 2.8 Co-immunoprecipitation (Co-IP)

NRVMs were lysed in IP buffer (150 mM NaCl, 50 mM Tris-HCl, pH 7.4, 1% NP-40, 1 mM EDTA) containing protease inhibitors. After protein quantification by BCA assay, 500 μg of total protein was incubated overnight at 4 °C with S100A10-or ANXA2-specific antibodies, followed by incubation with protein A/G agarose beads (Santa Cruz Biotechnology, United States) for 2 h at 4 °C. Immunoprecipitated proteins were eluted in SDS sample buffer and analyzed by Western blotting.

### 2.9 Statistical analysis

Data are presented as mean ± SEM from at least three independent experiments. Statistical significance was determined by two-tailed Student’s t-test for pairwise comparisons. Multiple comparisons were adjusted using Tukey’s *post hoc* test for ANOVA. Exact statistical tests are specified in each figure caption (e.g., two-way ANOVA with Tukey’s correction. A p-value <0.05 was considered statistically significant. All analyses were performed using GraphPad Prism 10.0.1.

## 3 Results

### 3.1 Expression of S100A10 in cardiomyocyte hypertrophy

**FIGURE 1:**
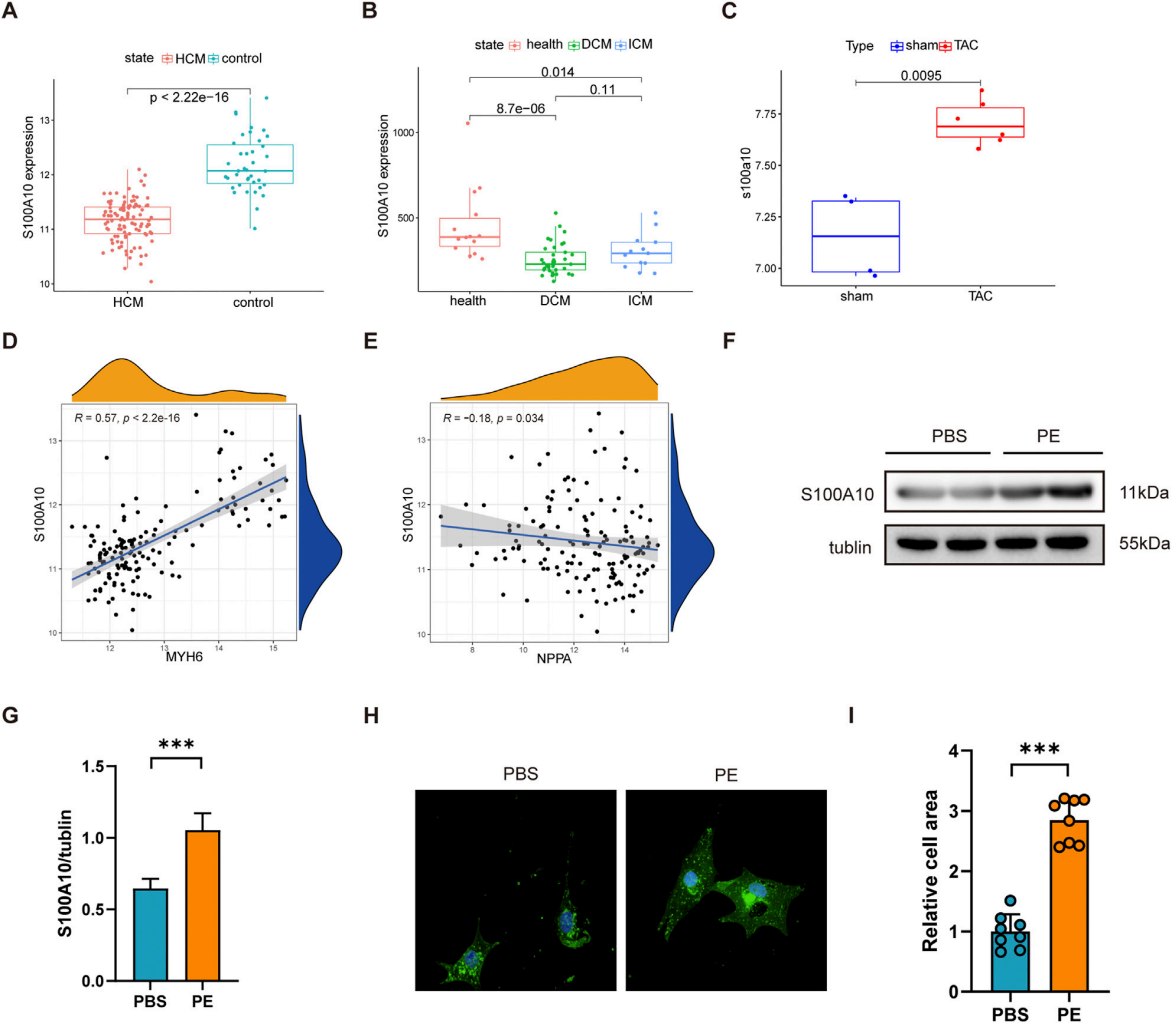
S100A10 expression is upregulated in cardiomyocyte hypertrophy models. Neonatal rat ventricular myocytes (NRVMs) were treated with phenylephrine (PE, 20 μM) or PBS (control) for 48 h **(A–C)** Box plots showing S100A10 mRNA expression levels in the left ventricle from three independent datasets (GSE5500, GSE116250, and GSE36961), analyzed by Wilcoxon test. **(D,E)** Spearman correlation analysis revealed significant associations between S100A10 and hypertrophy markers Nppa (weak correlation) and Myh6. **(F,G)** Western blot analysis revealed a significant upregulation of S100A10 protein levels in PE-induced hypertrophic NRVMs. (n = 3 biological replicates). **(H,I)** Representative wheat germ agglutinin (WGA) staining and quantification of cardiomyocyte cross-sectional area demonstrated PE-induced hypertrophy. Scale bar: 100 μm. Data are presented as mean ± SEM. *P < 0.05, **P < 0.01, ***P < 0.001. Abbreviations: MYH6, myosin heavy chain 6; NPPA, natriuretic peptide A; S100A10, S100 calcium-binding protein A10.

To comprehensively investigate S100A10 expression patterns in cardiac hypertrophy, we analyzed three independent gene expression datasets (GSE5500, GSE116250, and GSE36961) comprising both murine models of transverse aortic constriction (TAC)-induced hypertrophy and human samples of dilated cardiomyopathy (DCM) and ischemic cardiomyopathy (ICM). Our analysis revealed consistent and significant downregulation of S100A10 expression in human HCM, DCM, and ICM samples (p < 0.05). Interestingly, this pattern was reversed in TAC-induced hypertrophic mouse hearts, where S100A10 demonstrated significant upregulation (Figures 1A–C).

Further research has revealed that the expression of S100A10 is significantly positively correlated with MYH6 and a weak but significant negatively correlated with Natriuretic Peptide A (NPPA) (Figures 1D,E). Western blot analysis demonstrated a marked increase in S100A10 protein expression following PE stimulation in cardiomyocytes (Figures 1F,G). Morphological assessment through WGA staining revealed the expected hypertrophic response, with PE-treated neonatal rat ventricular myocytes (NRVMs) exhibiting significantly increased cross-sectional area compared to controls (Figures 1H,I).

Taken together, these results not only establish a reliable *in vitro* model of PE-induced cardiomyocyte hypertrophy characterized by S100A10 upregulation., but also highlight the differential regulation of S100A10 across distinct cardiomyopathy subtypes, suggesting potential subtype-specific roles in disease pathogenesis.

### 3.2 S100A10 knockdown exacerbates PE-Induced cardiomyocyte hypertrophy

**FIGURE 2:**
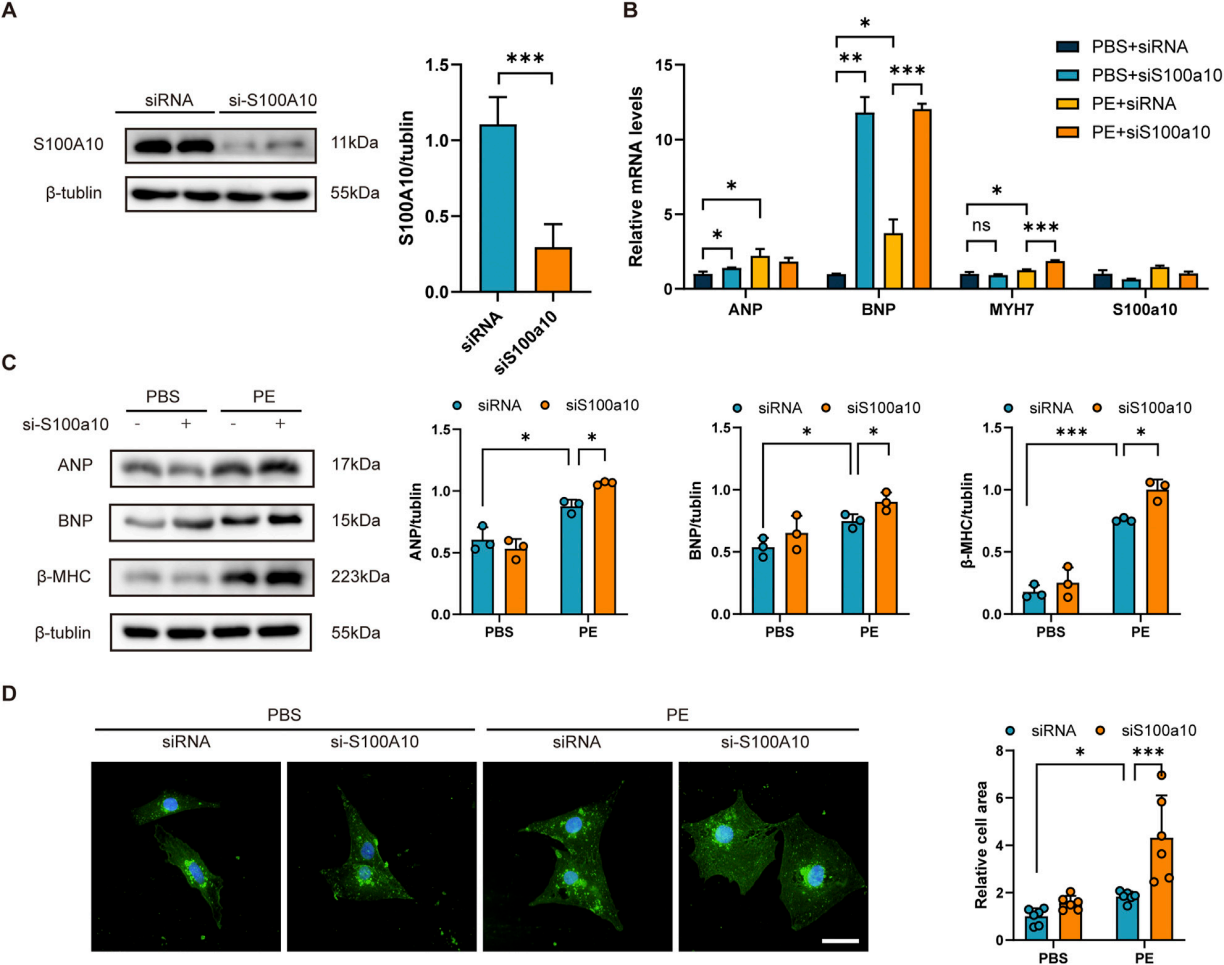
S100A10 silencing exacerbates PE-induced cardiomyocyte hypertrophy in NRVMs NRVMs were transfected with either control siRNA or S100a10-specific siRNA, followed by treatment with PE or PBS (control) for 48 h. (A) Western blot analysis and quantitative results demonstrating efficient knockdown of S100A10 protein in S100a10 siRNA-transfected NRVMs (n = 3 biological replicates). **(B)** Quantitative RT-PCR analysis showing mRNA expression levels of hypertrophic markers (Nppa, Nppb, and Myh7) in the indicated treatment groups (n = 6 independent experiments). **(C)** Representative Western blots and quantification of hypertrophic marker proteins (ANP, BNP, and β-MHC) in treated NRVMs (n = 3 biological replicates). **(D)** Representative wheat germ agglutinin (WGA) staining images and quantitative analysis of cardiomyocyte cross-sectional area (n = 6 independent experiments; scale bar = 100 μm). Data are presented as mean ± SEM. Statistical significance: *P < 0.05, **P < 0.01, ***P < 0.001 (two-way ANOVA with Tukey’s post-hoctest).

To investigate the functional role of S100A10 in cardiac hypertrophy, we performed siRNA-mediated knockdown of S100a10 in neonatal rat ventricular myocytes (NRVMs). Western blot analysis confirmed efficient silencing of S100A10 expression in transfected NRVMs (Figure 2A). Following PE stimulation, we observed significant upregulation of S100a10 at the mRNA level compared to PBS-treated controls (Figure 2B). Notably, PE treatment markedly upregulated the expression of hypertrophic markers Nppa (ANP), Nppb (BNP), and Myh7, an effect that was further potentiated by S100a10 knockdown (Figure 2B).

At the protein level, S100a10 deficiency synergistically enhanced PE-induced expression of ANP, BNP, and β-myosin heavy chain (β-MHC) (Figures 2B,C). Morphologically, S100a10 knockdown significantly augmented the PE-induced increase in cardiomyocyte cross-sectional area (Figure 2D), demonstrating its critical role in modulating hypertrophic growth.

### 3.3 S100A10 interacts with ANXA2 and activates the mTOR/4E-BPs pathway

**FIGURE 3:**
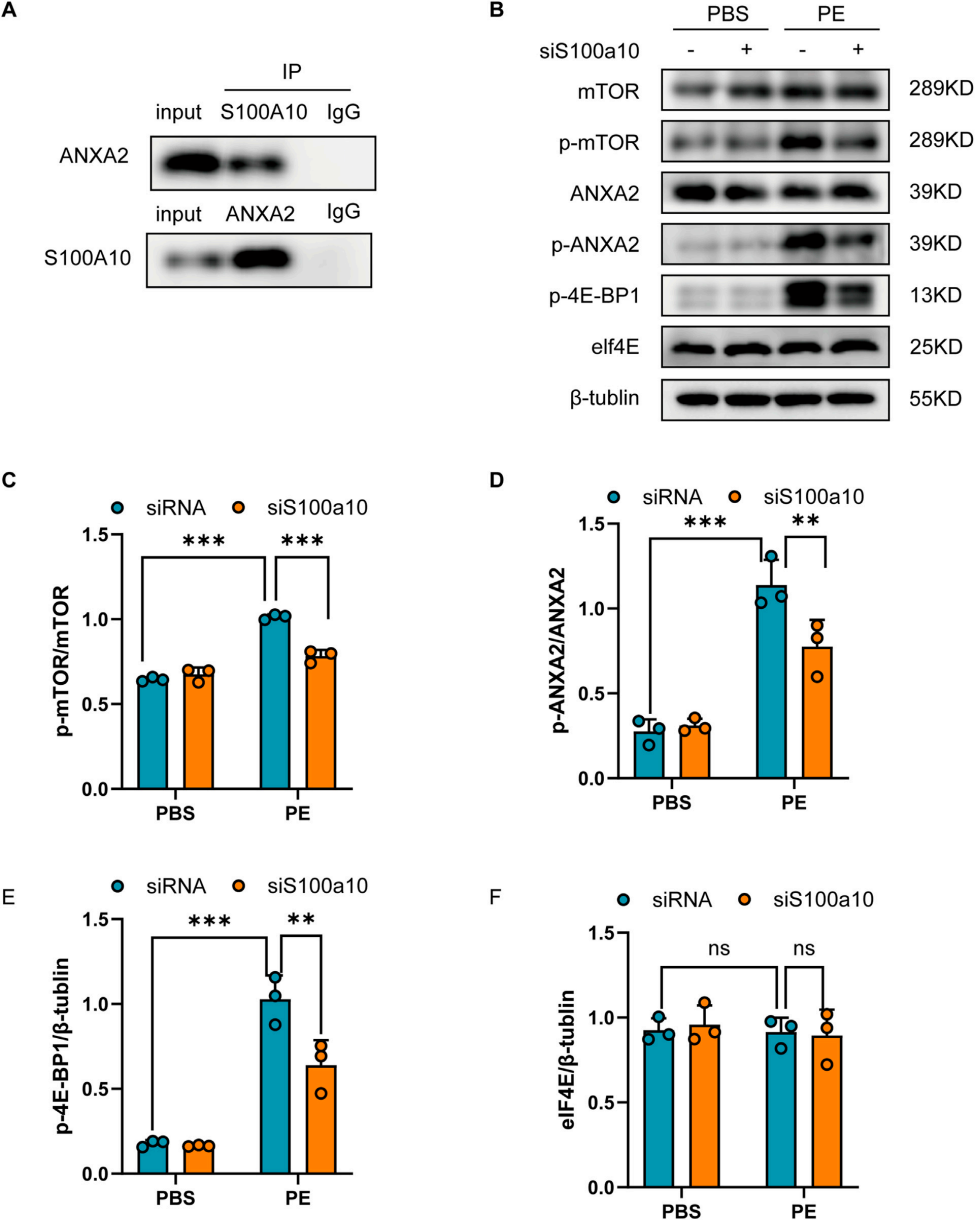
S100A10-ANXA2 interaction mediates mTOR/4E-BP1 pathway activation in hypertrophic cardiomyocytes Neonatal rat ventricular myocytes (NRVMs) transfected with control or S100A10-specific siRNA were treated with PE or PBS for 48 h. **(A)** S100A10-ANXA2 co-immunoprecipitation: Lysates immunoprecipitated (IP) with anti-ANXA2, anti-S100A10, or IgG control, then immunoblotted. **(B–F)** Representative western blots and quantitative densitometry of: mTOR pathway (mTOR, p-mTOR); ANXA2 signaling (ANXA2, p-ANXA2); Downstream targets (p-4E-BP1, eIF4E); β-tubulin was used as loading control (n = 3 biological replicates). Data represent mean ± SEM. Statistical significance: *P < 0.05, **P < 0.01, ***P < 0.001.

Existing literature has well documented that S100A10 predominantly exists in a heterotetrameric complex with its binding partner annexin A2 (ANXA2), termed the AIIt (annexin II tetramer) complex. Notably, elevated S100A10 expression has been demonstrated to facilitate ANXA2 phosphorylation at Tyr23 and Ser25 residues, which subsequently triggers activation of the mechanistic target of rapamycin (mTOR) signaling cascade (Li et al., 2020). To investigate whether this phenomenon also occurs in the PE-induced cardiomyocyte hypertrophy model, we performed co-immunoprecipitation (Co-IP) assays using protein extracts from PE-treated NRVMs. The results confirmed that S100A10 interacts with ANXA2 under hypertrophic conditions (Figure 3A).Based on existing evidence, we propose a mechanistic model wherein S100A10 modulates mitochondrial function through two interconnected pathways: (1) regulation of ANXA2 phosphorylation status, and (2) subsequent activation of the mTOR/4E-BP1 signaling axis. As expected, Western blot results demonstrated that PE stimulation significantly increased the phosphorylation levels of mTOR, ANXA2, and 4EBP1, while knockdown of S100A10 further attenuated these phosphorylation events, demonstrating its regulatory role in this signaling pathway (Figures 3B–F).

### 3.4 Knocking down of S100a10 exacerbates PE-induced mitochondrial respiratory dysfunction

**FIGURE 4:**
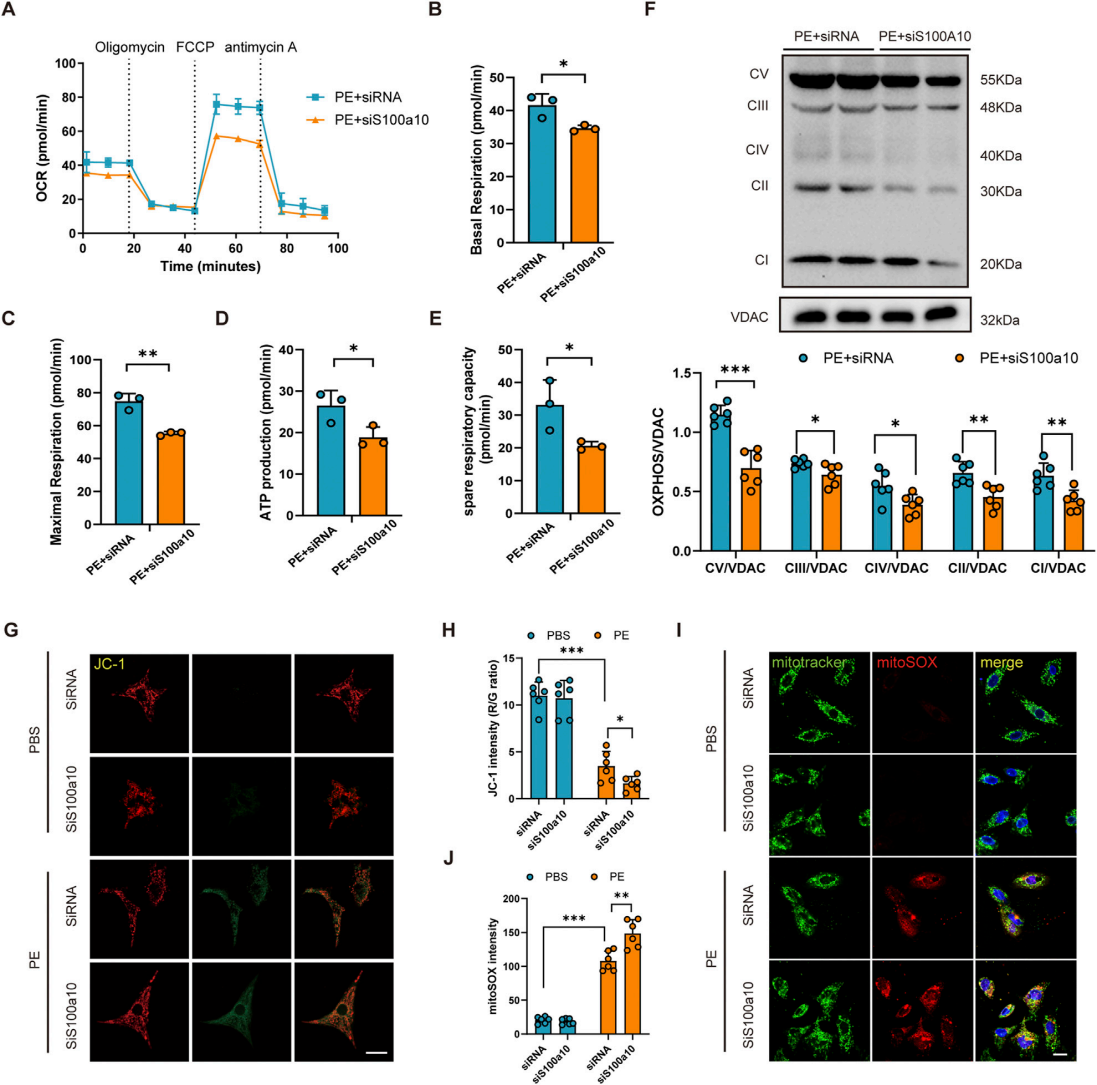
S100a10 regulates mitochondrial function and oxidative stress in PE-induced cardiomyocyte hypertrophy NRVMs were transfected with control siRNA or S100a10-specific siRNA, followed by treatment with PE or PBS (control) for 48 h. **(A)** Real-time measurement of oxygen consumption rate (OCR) using the Seahorse XFe24 Analyzer. **(B–E)** Quantitative analysis of mitochondrial respiration parameters: basal respiration, maximal respiration, spare respiratory capacity, and ATP production. **(F)** Western blot analysis of mitochondrial electron transport chain (ETC.) complexes (CI-V) and Voltage-Dependent Anion Channel (VDAC) (loading control). **(G,H)** Assessment of mitochondrial membrane potential by JC-1 staining (red/green fluorescence ratio) with quantitative analysis. **(I,J)** Measurement of mitochondrial reactive oxygen species (ROS) using MitoSOX Red and MitoTracker Green co-staining with quantitative analysis (scale bar = 100 μm). Data represent mean ± SEM from 3-5 independent experiments. Statistical significance: *P < 0.05, **P < 0.01, ***P < 0.001.

Seahorse analysis demonstrated that S100A10 knockdown in NRVMs significantly impaired mitochondrial function following PE stimulation, as evidenced by reduced oxygen consumption rate (OCR), ATP production, and maximal respiration (Figures 4A–E). Consistent with these observations, S100A10-deficient cardiomyocytes exhibited a marked decrease in mitochondrial complex protein levels, particularly Complex V (Figure 4F). Furthermore, PE stimulation led to a significant decline in mitochondrial membrane potential, which was further exacerbated by S100A10 knockdown (Figures 4G,H). Notably, mitoSOX staining revealed a synergistic increase in mitochondrial ROS production in si-S100A10 cardiomyocytes upon PE stimulation (Figures 4I,J).

## 4 Discussion

In this study, we investigated the role of S100A10 in phenylephrine (PE)-induced cardiomyocyte hypertrophy, focusing on mTOR pathway activation and its downstream impact on mitochondrial function. Our findings demonstrate that S100A10, via interaction with ANXA2, modulates mTOR/4E-BP signaling activation, ultimately inducing mitochondrial dysfunction and exacerbating cardiomyocyte hypertrophy. This work provides the first evidence that S100A10 modulates mitochondrial function through regulating respiratory chain protein levels—a process potentially mediated by mTOR/4E-BP activation.

S100 proteins are a family of highly acidic calcium-binding proteins, some of which are highly expressed in heart tissue. A growing body of evidence points to their involvement in fundamental cellular processes, including the regulation of mTOR signaling pathways and mitochondrial function, forming an independent area of investigation. Defects in intracellular calcium (Ca^2+^) processing are associated with the pathogenesis of heart failure. The Ca^2+^ sensor protein S100A1 was found to be significantly downregulated in human end-stage HF (Remppis et al., 1996). Through S100A1 gene targeting therapy, even a modest change in S100A1 protein can also prove *in vitro* treatment of human cardiac failure (Brinks et al., 2011). Due to its molecular characteristics, S100A1 has become an attractive target for gene-targeted HF therapy in various *in vivo* HF models. Two members of the S100 Ca (2+) binding protein family, S100A2 and S100A6, have high sequence homology and differ in their effects on cardiac Ca (2+) processing and contractility. Cardiac gene expression S100A2 significantly enhances systolic and diastolic performance of rodent and canine cardiomyocytes, mimicking the functional role of its cardiac congener S100A1. In contrast, cardiac expression of S100A6 had no significant effect on contractility and Ca (2+) processing. These data reveal new differential effects of the S100 protein on cardiomyocyte performance, which may be useful in applications of diseased heart muscle (Wang et al., 2014). The transfer-related protein S100A4 is expressed in a variety of cells, including fibroblasts, and is considered a marker of fibrosis in several organs. In the Dahl-rat hypertensive heart disease model, S100A4 is upregulated in hypertrophic myocardia and further activated during the transition to centripetal heart failure (HF). *In vitro* cardiac fibroblasts, S100A4 knockdown significantly inhibited cell proliferation and collagen expression. In mice with cardiac remodeling induced by TAC, S100A4-KO mice showed reduced interstitial fibrosis, reduced myofibroblasts, and inhibition of left ventricular collagen and pro-fibrotic cytokine expression, suggesting that blocking S100A4 may have therapeutic potential for cardiac hypertrophy and HF by alleviating cardiac fibrosis (Tamaki et al., 2013). In recent years, the neutrophil-derived S100 calcium-binding protein A8/A9 (S100A8/A9) has attracted more and more attention as an important early warning protein of cardiovascular diseases, which has a multi-aspect impact on cardiovascular diseases such as atherosclerosis, myocardial infarction, myocardial ischemia/reperfusion injury and heart failure, and is considered as a key biomarker for the diagnosis and prediction of various cardiovascular diseases (Sun et al., 2024). In a prospective study, S100A8/A9 levels on day 1 of HF in patients after ACS reperfusion therapy accurately classified patients at different HF risk and could serve as a powerful tool for HF risk prediction and treatment guidance (Ma et al., 2025). All the above studies have confirmed that S100a family is closely related to the occurrence and development of cardiovascular diseases. However, S100A10, as a member of the S100a family, has been less studied in cardiovascular diseases. In a meta-analysis of blood pressure and hypertension whole-blood gene expression profiles, six blood pressure-related transcripts, including S100A10, were identified (Huan et al., 2015). This prompted us to consider whether the process of myocardial remodeling induced by pressure overload is related to S100A10. In the first place, we found that S100a10 was upregulatde during PE-induced cardiomyocyte hypertrophy according to differentially expressed genes and molecular biological validation (Figure 1). Although S100A10 is downregulated in the human DCM/ICM dataset, its upregulation in TAC mice and NRVMs treated with PE may reflect model-specific pathophysiological stages. Human cardiomyopathy is often accompanied by a chronic fibrotic microenvironment, representing end-stage heart failure (Frangogiannis, 2021; Cojan-Minzat et al., 2021; Schlittler et al., 2023; Chang et al., 2023). In contrast, the mouse TAC model focuses more on acute pressure overload with compensatory hypertrophy (Bacmeister et al., 2019). There are differences in the pathological microenvironment between the two. The mechanical stress induced by TAC surgery may activate compensatory pathways, masking the true regulatory pattern of S100A10 in human failing hearts. PE-induced NRVMs simulate acute adrenergic stress, which has a time course difference from the chronic process of progressive myocardial remodeling in humans. While dysregulation of S100 proteins are implicated in heart failure, recent research has revealed broader mechanistic links: specific S100 proteins are now recognized for their emerging roles in modulating mitochondrial homeostasis (Wang et al., 2024; Wu et al., 2023; Pei et al., 2024; Zhang et al., 2023) and mTOR signaling (Fan et al., 2023; Teng et al., 2021; Zhou et al., 2024), highlighting functions that extend beyond cardiovascular pathology. Regarding the regulatory role of S100A10 in the mTOR/4E-BP pathway identified in this study, we propose a mechanism influenced by both disease stage and species-specific factors. During early compensatory hypertrophy, activation of the mTOR/4E-BP pathway may enhance the translation of protective proteins such as S100A10. In contrast, under the chronic stress conditions of end-stage heart failure, translational suppression or increased protein degradation may prevail. Furthermore, differences in translational regulatory elements or mTOR signaling components between humans and rodents could affect the translation efficiency and stability of S100A10 mRNA. Notably, since the mTOR/4E-BP pathway is a central regulator of protein synthesis, variations in its activity across species or disease stages may directly modulate S100A10 protein expression—a dimension not captured by transcriptomic data alone. Thus, the observed expression differences likely originate from translational rather than transcriptional regulation. Future work should integrate direct measurements of protein synthesis rates (e.g., puromycin labeling) with cross-species models to further elucidate the translational control of S100A10 during cardiac remodeling. The correlation analysis results between S100a10 and MYH6 indicate that S100a10 may have a potential protective effect in regulating the balance between compensatory cardiac growth and stress response, while S100a10 shows a weak but significant negative correlation with NPPA (R = −0.16, P = 0.034). This weak correlation may reflect that the two are jointly regulated by other pathological factors, which needs to be further verified in combination with functional experiments. In subsequent *in vitro* experiments, we demonstrated that S100A10 knockdown alleviates PE induced cardiomyocyte hypertrophy. At present, there is a lack of evidence on the role of S100A10 in ischemic heart disease. Whether S100A10 can be used as a target tool for HF risk prediction and treatment guidance remains to be confirmed by further studies.

ANXA2 is a membrane binding protein involved in cardiomyocyte proliferation, apoptosis, and morphological changes (Kayejo et al., 2023). Many proteins have been found to interact with ANXA2 to produce biological effects. For example, previous studies have shown that S100A10 and ANXA2 interact significantly (Banerjee et al., 2015), which suggests that we should pay attention to the possible effects of S100A10 and ANXA2 on cardiomyocyte hypertrophy. Consistent with previous studies, we also detected the interaction between S100A10 and ANXA2 in NRVMs, and this interaction tended to be enhanced under the stimulation of PE, which may further confirm the role of ANXA2 in cardiac hypertrophy. Recent studies have also shown that ANXA2 phosphorylation plays a key role in activating the mTOR pathway, which is critical for regulating processes such as autophagy and protein synthesis (Ji et al., 2025). Similar to previous findings, we found that the interaction of S100A10 and ANXA2 activates the mTOR pathway, leading to downstream effector mitochondrial dysfunction (Figure 4). In this study, we detected that S100A10 downregulation inhibited mitochondrial oxidative phosphorylation. Interestingly, however, we also found downregulation of mitochondrial respiratory chain protein expression levels, implicating that S100A10 may also affect mitochondrial oxidative phosphorylation by regulating mitochondrial respiratory chain protein synthesis. Previous studies have pointed out that protein synthesis mediated by mTOR/4EBPs pathway plays an important role in cardiac hypertrophy (Morita et al., 2013; Wang et al., 2000). Here, we also detected the activation of mTOR/4EBPs pathway. While mTOR/4E-BP activation suggests translational control, direct evidence (e.g., puromycin incorporation) is pending. Future studies will quantify nascent protein synthesis in S100A10-deficient cardiomyocytes. Previous studies demonstrated S100A10-ANXA2 interaction activates AKT/mTOR signaling and enhances glycolytic metabolism in osteosarcoma cells (Ling and Lu, 2022). Based on cross-disease molecular parallels, we hypothesize that S100A10 may possess metabolic regulatory functions. However, whether S100A10’s impact on mitochondrial function in phenylephrine-induced cardiomyocyte hypertrophy is mediated through glycolytic metabolism requires experimental confirmation.

While this study offers valuable insights into the mechanisms by which S100A10 and ANXA2 interact to regulate PE-induced myocardial hypertrophy, several limitations exist. First, while the PE-induced NRVMs *in vitro* model effectively recapitulates early-stage phenotypes of cardiomyocyte hypertrophy, the absence of systematic validation in pressure-overload animal models (e.g., TAC-operated mice) remains a significant constraint. Future investigations should comprehensively evaluate the functional consistency of S100A10 at the organ level through *in vivo* approaches. Second, the interaction between S100A10 and ANXA2 may involve other complex molecular mechanisms that require further exploration and the clinical application of S100A10 and ANXA2 in cardiovascular diseases requires evaluation through larger-scale clinical studies. Lastly, the use of JC-1 staining for assessing mitochondrial membrane potential has limitations, and future studies will employ methods such as TMRE or TMRM for further validation.

## 5 Conclusion

This study explores the role of the interaction between S100A10 and ANXA2 in PE-induced myocardial hypertrophy and demonstrates that S100A10, through activation of the mTOR/4EBPs pathway, may regulates mitochondrial protein synthesis and mitochondrial function, thereby alleviating PE-induced myocardial hypertrophy. These findings provide new perspectives on the pathogenesis of cardiovascular diseases and offer a theoretical basis for future therapeutic strategies.

## Data Availability

The datasets presented in this study can be found in online repositories. The names of the repository/repositories and accession number(s) can be found in the article/Supplementary Material. All analysis code has been deposited in a publicly accessible GitHub repository at https://github.com/xfx2025xfx/20250728.
