# Supplementary material for: S100A10 knockdown exacerbates phenylephrine-induced cardiomyocyte hypertrophy via modulating mitochondrial oxidative phosphorylation
Source: Front Genet. 2025 Oct 22;16:1610008. doi: 10.3389/fgene.2025.1610008 (PMC12585156; doi:10.3389/fgene.2025.1610008)

Supplementary Figure S1: Protein-protein interaction (PPI) network analysis using the STRING database（https://cn.string-db.org/）on DEGs from the GSE5500


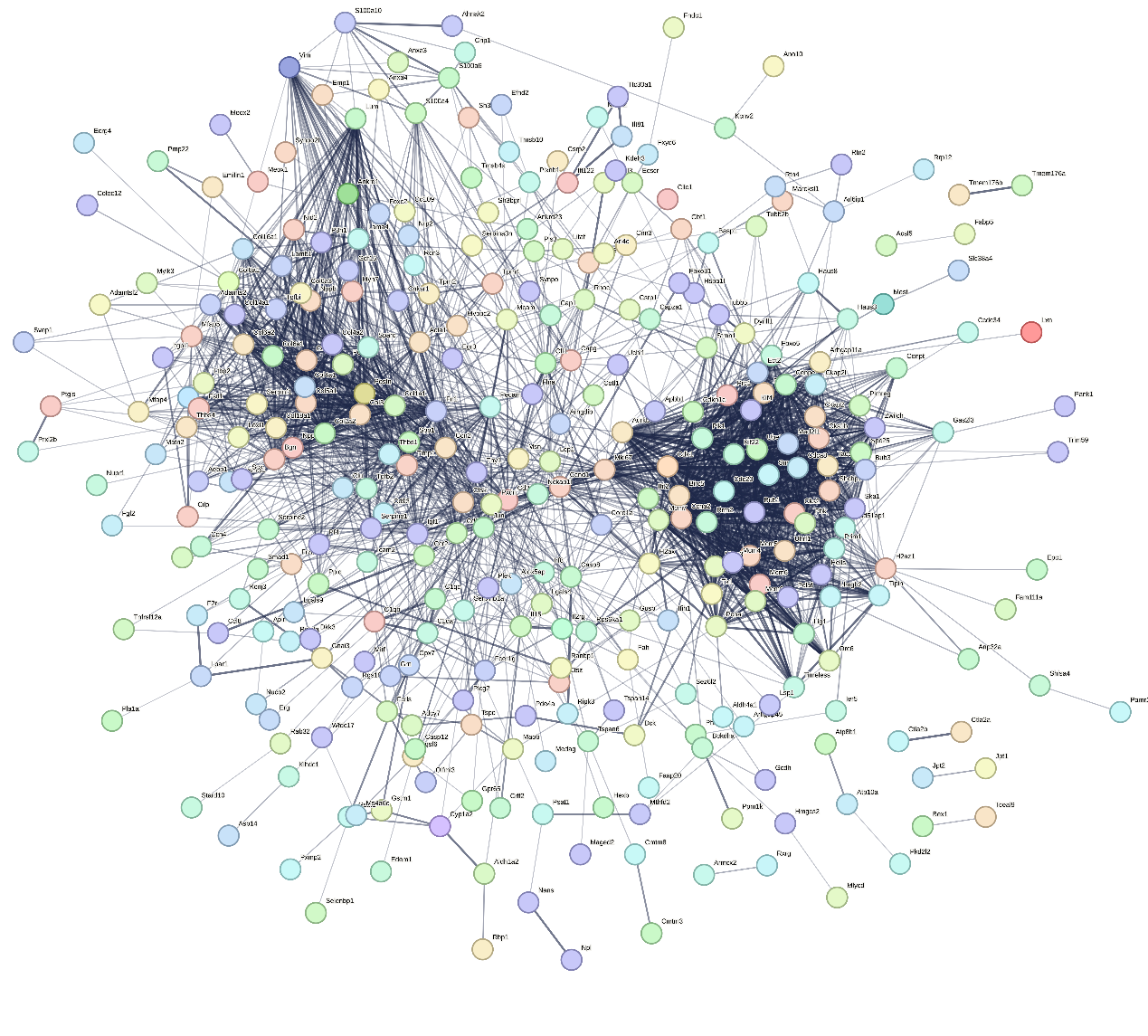


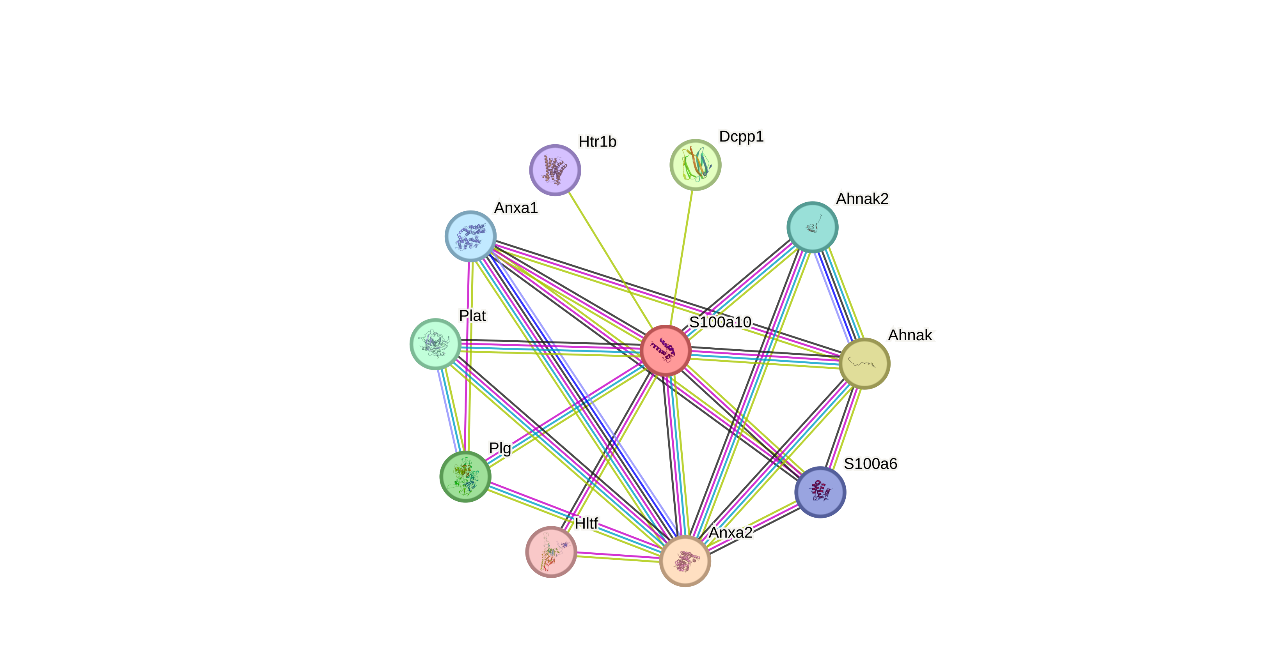


Supplementary Figure S2：Heatmap of the top 40 DEGs (based on adjusted p-value and fold change) in TAC versus sham groups (GSE5500).


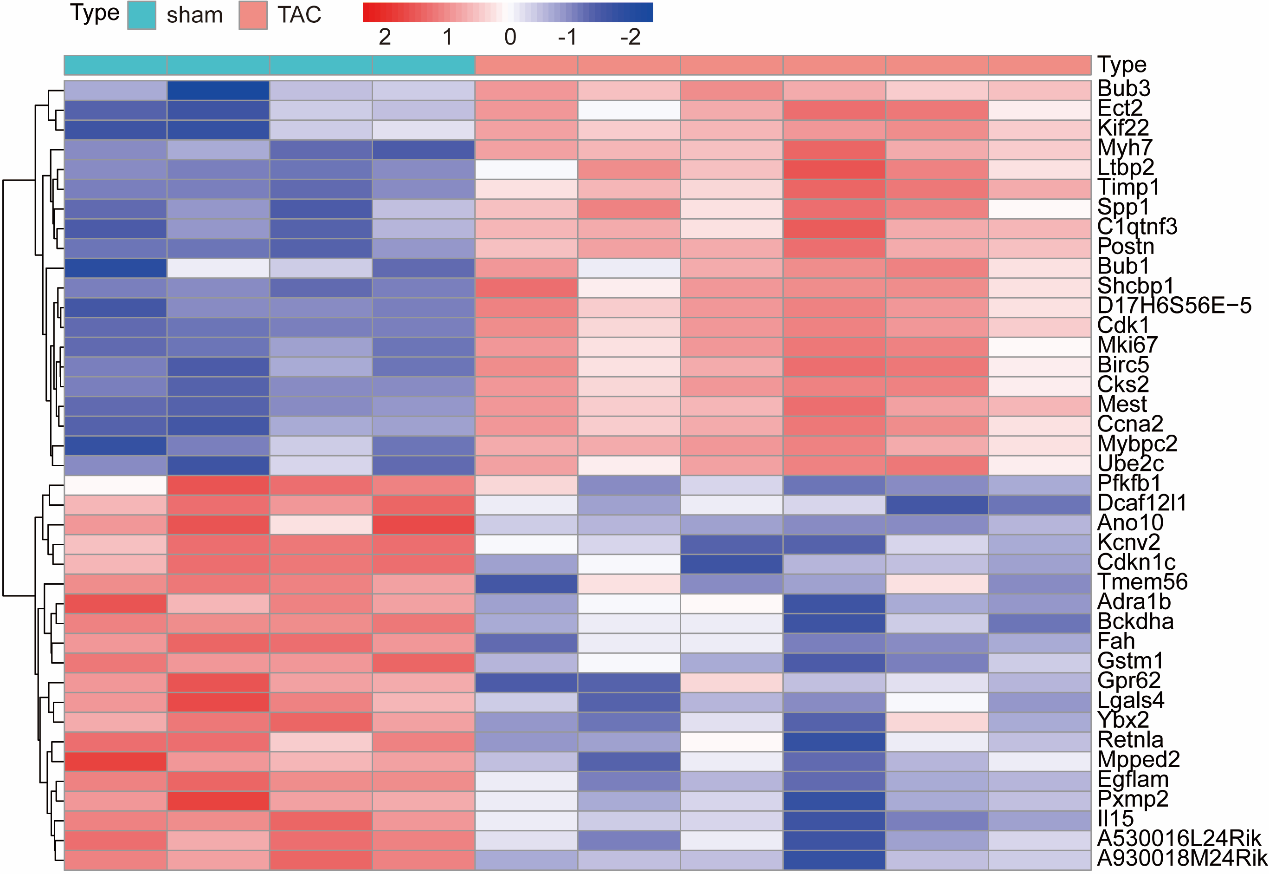


Supplementary Figure S3：Box plots showing anxa2 mRNA expression levels in the left ventricle from GSE5500 datasets analyzed by Wilcoxon test


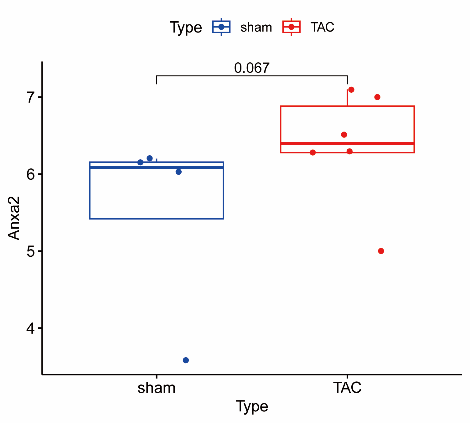


Supplementary Figure S4：GSEA was performed on transcriptomic data from hypertrophic cardiomyopathy models stratified by S100A10 expression levels. Enrichment plots are shown for three hallmark gene sets that were significantly enriched:


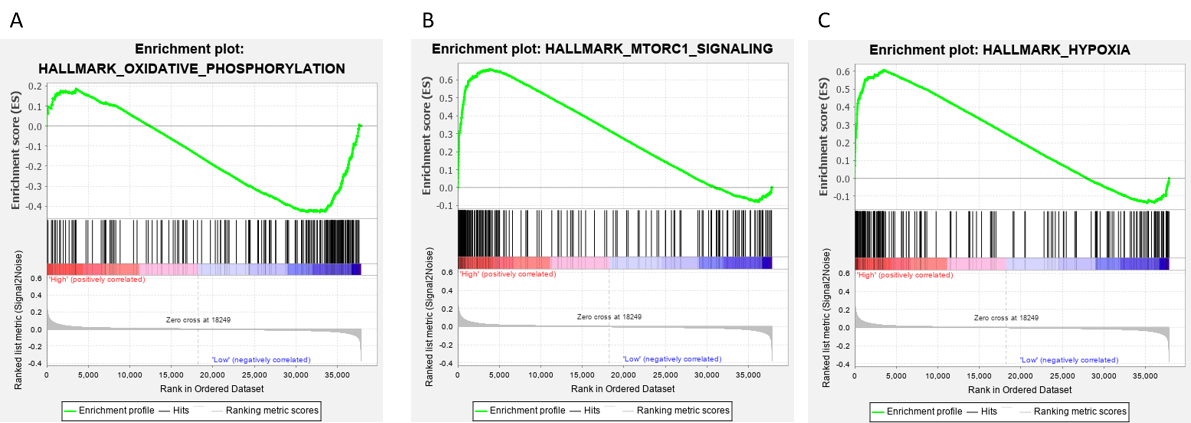

Supplement: Supplementary file 2 [file Supplementaryfile1.doc]
